# Assessing the Impact of Removable Prosthetic Restorations on Nutritional Habits in Edentulous Patients Following Surgery for Head and Neck Cancer

**DOI:** 10.3390/nu17091483

**Published:** 2025-04-28

**Authors:** Beata Sawczuk, Suresh Nayar, Paweł Szutko, Teresa Sierpińska

**Affiliations:** 1 Department of Prosthodontics, Medical University of Białystok, ul Kilińskiego 1, 15-089 Białystok, Poland; 2 Institute for Reconstructive Sciences in Medicine, University of Alberta, 1W-02, 16940 87 Ave NW, Edmonton, AB T5R 4A3, Canada; snayar@ualberta.ca; 3 Medical University of Białystok, ul Kilińskiego 1, 15-089 Białystok, Poland; pawel.sz2904@gmail.com

**Keywords:** nutritional habits, malnutrition, surgical management, head and neck cancer, edentulous

## Abstract

Background: Head and neck cancers (HNCs) and their surgical treatment can result in significant functional deficits including impaired masticatory function, dysphagia and dysgeusia, among others. These contribute to nutritional deficits weakening immune responses, increased post-surgical infections and complications. Aim: This study assesses the impact of removable prosthetic restorations on nutritional habits in edentulous patients who have undergone surgery for head and neck cancer. Materials and methods: This study included 44 post-surgical oncology patients and 20 healthy edentulous patients who served as controls. All patients received removable acrylic complete prostheses. Controls received maxillary and mandibular complete prostheses and HNC patients received post-resection complete maxillary and mandibular prostheses. Nutritional intake was assessed through a 24 h dietary recall and the Food Frequency Questionnaire administered before prosthetic treatment and 6 weeks and 3, 6 and 12 months after the provision of removable prosthetic restorations. Results: This study found that both patient groups maintained consistent meal frequency, with the study group exhibiting stable food intake over time. The intake of various food items fluctuated post prosthesis delivery in both groups, with an initial decline followed by partial recovery. Statistically significant differences were observed in food preferences; however, diet variations between and within the groups were not statistically significant. Conclusions: This study found that the use of removable prosthetic restoration in surgically managed edentulous head and neck cancer patients and edentulous controls showed no significant differences apart from certain food preferences and diet variations. A prolonged adaptation period was observed highlighting the need to include clinical dietitians to support the patients.

## 1. Introduction

Head and neck cancer (HNC) is the sixth most common malignant neoplasm and accounts for roughly 4.6% of global cancer deaths. Globally, head and neck cancers most often occur in patients after 50 years of age, and they affects men twice more frequently than women [1]. There is a rise in the global incidence of HNC, particularly in younger populations and partly attributed to changes in lifestyle factors such as increased use of alcohol and tobacco. There is also growing prevalence of human papillomavirus (HPV)-related oropharyngeal cancer, which is estimated to overtake tobacco as the major contributor of HNC [2,3]. The common risk factors for HNC includes tobacco use, alcohol, poor oral health, occupational exposure, radiation exposure, diet and nutrition, immunosuppression, ethnicity and genetic predisposition, HPV and Epstein–Barr virus infection [4].

HNC and its treatment can have long-term adverse effects and can impact patients in various ways [4]. Surgical removal of the cancerous tissues can lead to anatomical defects resulting in aesthetic and physiologic or functional deficits and psychological challenges. The functional deficits created by surgical treatment are due to the highly sensitive and complex anatomical region, which can result in the patient being unable to intake food orally [5]. The functional deficits can include loss of dentition, altered denture foundation, xerostomia, trismus, orodynia, dysphagia. dysgeusia and aspiration. This can result in nutritional deficit or, in extreme cases, malnutrition in these patients [6]. Malnutrition weakens therapeutic and immune responses, increasing the risk of infections and post-surgical complications. It can also lead to treatment interruptions, greater financial burdens, reduced functional abilities and ultimately lower quality of life.

The reconstruction of lost tissues and teeth depends on the surgical procedures and is based on tumour location, resection size and type as well as the number of teeth preserved or lost. The choice of restoration of missing dentition can depend on many factors, including its affordability. The most common modality of restoration in these patients is the use of post resection removable prostheses, which, despite their many limitations, have advantages with widespread use and acceptance by patients [7].

Functionally, conventional removable prostheses have limitations with regard to chewing efficiency. They require greater chewing cycles for food comminution and hence take more time and could affect taste and choice of food along with difficulties in swallowing. The reduction in chewing efficiency and its attendant sequelae can limit the intake of key nutrients and affect patients’ general health and quality of life. A search of the literature has revealed a paucity of studies conducted on nutrition and food preferences in patients provided with post-resection removable prostheses [8,9,10]. Will the use of removable dentures by cancer group improve their nutritional status? Will the nutritional preferences of the study group change thanks to the prostheses? Do cancer patients need more time to adapt to the new prostheses?

This study aims to assess the impact of removable prosthetic restorations on the nutritional habits in edentulous patients, following surgery for head and neck cancer.

## 2. Materials and Methods

This study was conducted between 2022 and 2024. The proposed study was submitted to the Local Bioethics Committee at the Medical University of Białystok (MUB), Poland, and ethical approval was obtained (APK.002.115.2024). All the recruited patients were provided with an information leaflet of the research, and informed consent was obtained by signature on a consent form.

A total of 64 patients (39 females and 25 males) of the Dental Prosthetics Department (DPD), MUB, Poland, were included in this study. They were divided into two groups (study group—44; control group—20). The mean age of the subjects was 64.523.

**Figure 1:**
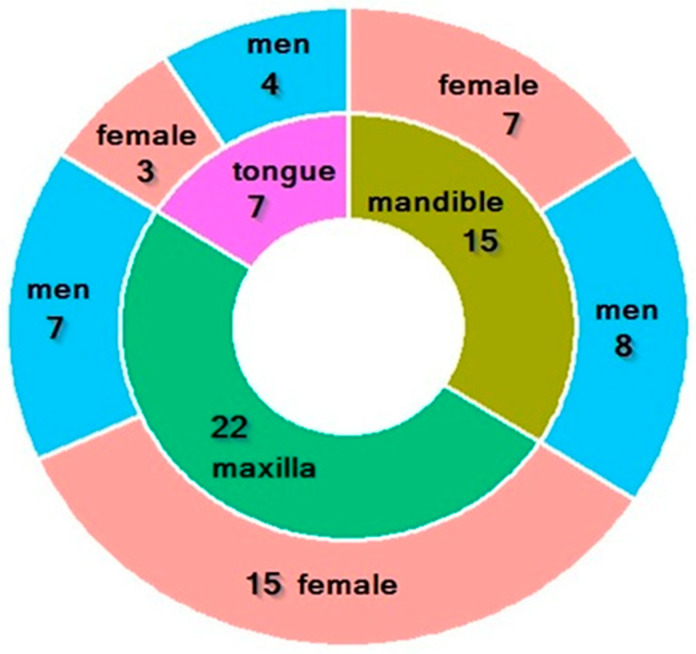
Characteristics of the study group. Green—22 patients with maxilla cancer (7 men, 15 females); olive—15 patients with mandible cancer (8 men, 7 females); purple—7 patients with tongue cancer (7 men, 3 females).

The study group (44, male—19; female 25) was composed of edentulous patients who had been operated on at the DPD due to head and neck cancer and had been referred to the DPD for prosthetic treatment. They underwent surgery due to tongue cancer (7), maxillary cancer (22) and mandibular cancer (15) (Figure 1). Patients with stage T1 N0 M0 and T2 N0 M0 were qualified for the study, in addition to those with postoperative defects Type I and II according to Dreher’s classification.

The control group (20; male—6; female—14) was made up of healthy edentulous individuals.

All patients in both the study and control groups completed the study.

The inclusion and exclusion criteria are presented in Table 1 and Table 2.

The prosthetic foundation for patients in both groups was evaluated clinically. The study group was provided with post-resection removable maxillary and/or mandibular complete prostheses, and the control group was provided with removable maxillary and mandibular complete prostheses. Patients from both groups had never had dentures before. The evaluations and the provision of the removable prostheses were carried out by one clinician. The removable prostheses were made as part of public insurance, which covers all citizens in Poland.

A combined quantitative and qualitative approach was used to assess nutritional status. Data collection for the quantitative part involved a 24 h dietary recall, evaluating the consumption of food, beverages and specific food product groups using the Food Frequency Questionnaire [11]. The methodology adhered to the Centre for Food and Nutrition guidelines [12]. The 24 h recall was conducted three times per participant on non-consecutive weekdays (Monday, Wednesday and Friday) to enhance accuracy. All patients were carefully instructed on how to complete the questionnaire. Patients completed the questionnaire an ongoing basis before the visit. Portion sizes were determined by participants using a standardised photographic food atlas [13]. Questionnaires were completed before start of prosthetic treatment and 6 weeks and 3, 6, 12 months post prosthetic treatment.

The 24 h dietary recall questionnaire information was used to assess diet composition including their energy values. The 24 h dietary interview method is a retrospective, questionnaire-based, qualitative–quantitative method that provides accurate information on the amount and type of food and beverages consumed. The method determines current consumption. The obtained data were entered into DIETA 6.0 software developed by the Institute of Food and Nutrition in Poland, which made it possible to estimate the intake of energy and the main nutrients, i.e., protein, fat and carbohydrates, as well as selected vitamins and minerals (Dieta 6, NIZP PZH-PIB, PZH, Warsaw, Poland).

For the purposes of this study, medical data were additionally collected from each patient’s medical history regarding comorbidities, used medications and the complications of treatment. A history was taken, based on which the presence of different symptoms accompanying the underlying disease and its treatment was noted.

The results were subjected to statistical analyses. The mean (M), standard deviation (SD), median (Me), minimum (min.) and maximum (max.) values were used for descriptive statistics. The Mann–Whitney test was used to compare nutrient intake across the specified time points based on the 24 h dietary interview between the two groups. The Pearson’s chi-square test of independence and the two-proportion test were applied to assess the intake frequency of the selected food groups to compare incidence rates between the two groups. A post hoc test for the Friedman test was used to analyse the intake frequency of the selected food products over time in both the groups. Cran R statistical software and the scmamp package were used for the calculations. A *p*-value of less than 0.05 was considered statistically significant.

## 3. Results

Both groups of patients consumed five meals a day, with food intake amongst the study group remaining relatively stable over time.

### 3.1. Energy Value

**Figure 2:**
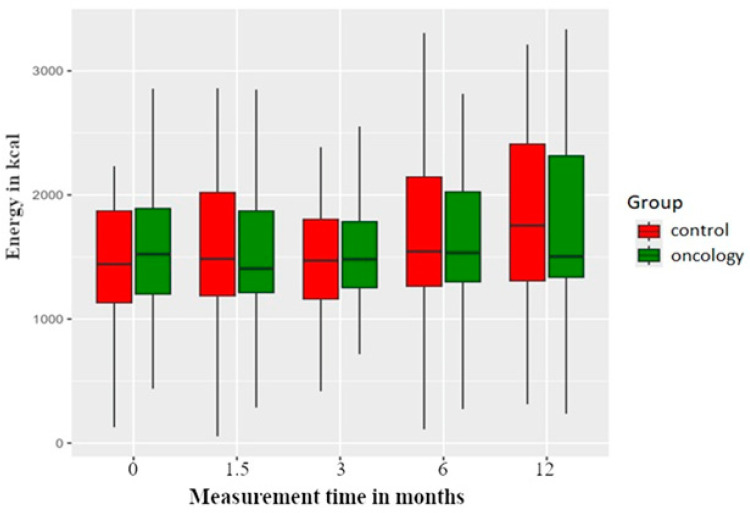
The energy value of meals in the study and control groups across all measurement periods. Time 0—before prosthetic treatment; Time 1.5—6 weeks (1.5 months) post prosthetic treatment; Time 3—3 months post prosthetic treatment; Time 6—6 months post prosthetic treatment; Time 12—12 months post prosthetic treatment.

The energy content of meals in the study group declined after six weeks of prosthesis use, although it remained stable at that level in subsequent measurements. For the control group, there was an increase in the energy content values at 12 months in comparison with pretreatment values. However, none of these changes were statistically significant within or between the groups (Figure 2).

### 3.2. Macronutrients

**Figure 3:**
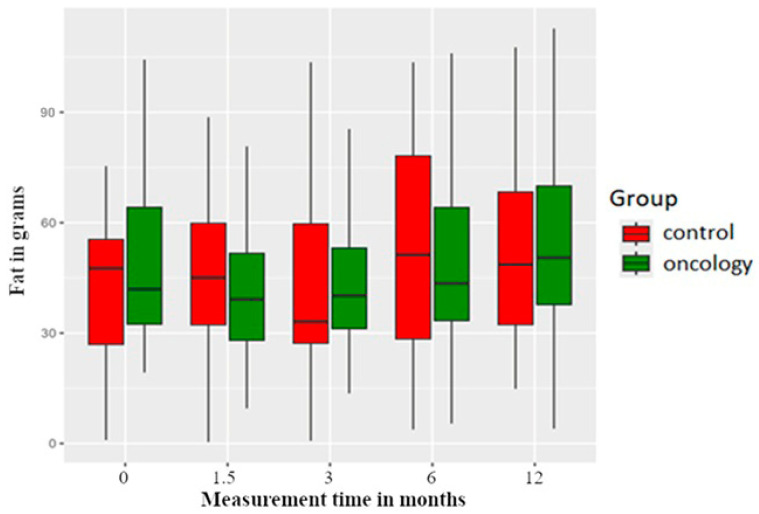
Fat consumption in the study and control groups across all measurement periods. Time 0—before prosthetic treatment; Time 1.5—6 weeks (1.5 months) post prosthetic treatment; Time 3—3 months post prosthetic treatment; Time 6—6 months post prosthetic treatment; Time 12—12 months post prosthetic treatment.

The study group consumed less fat than the control group before the treatment. However, fat intake decreased in both groups in the 6 weeks and 3 months post prosthesis delivery, followed by an increase in both groups for the 6- and 12-month measurements. However, the differences were not statistically significant between or within the groups (Figure 3).

**Figure 4:**
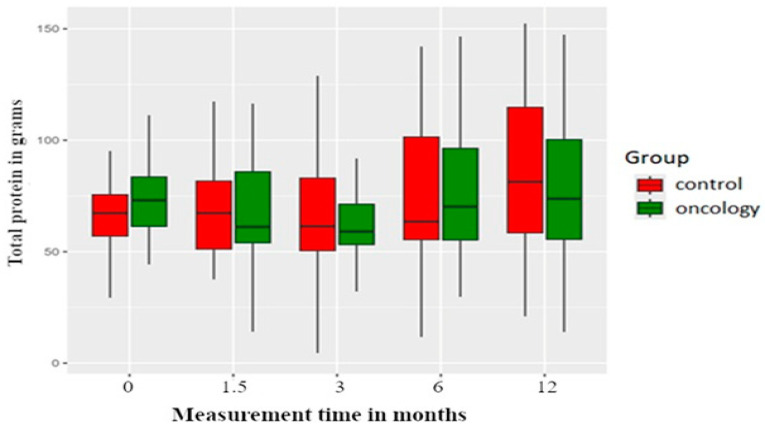
Total protein consumption in the study and control groups across all measurement periods. Time 0—before prosthetic treatment; Time 1.5—6 weeks (1.5 months) post prosthetic treatment; Time 3—3 months post prosthetic treatment; Time 6—6 months post prosthetic treatment; Time 12—12 months post prosthetic treatment.

**Figure 5:**
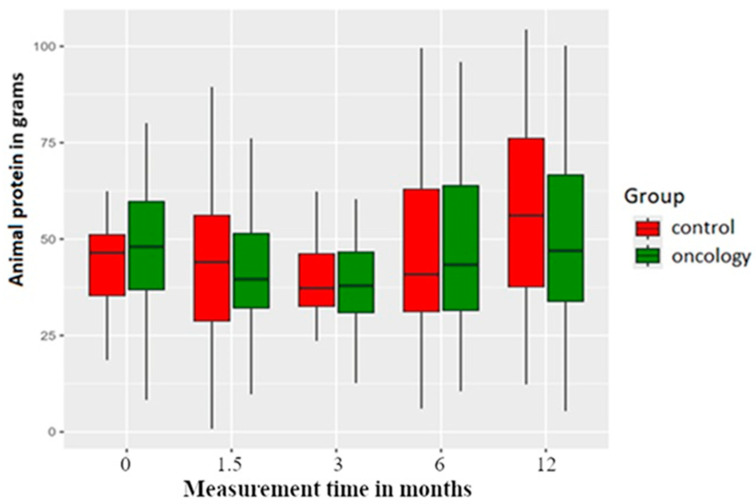
Animal protein consumption in the study and control groups across all measurement periods. Time 0—before prosthetic treatment; Time 1.5—6 weeks (1.5 months) post prosthetic treatment; Time 3—3 months post prosthetic treatment; Time 6—6 months post prosthetic treatment; Time 12—12 months post prosthetic treatment.

Total and animal protein consumption was higher in the study group before the treatment. Six weeks and three and six months post prosthesis delivery, there was reduction in protein intake in both groups, with an increase noted at the twelve-month measurement. However, none of these changes were statistically significant between or within the groups (Figure 4 and Figure 5).

**Figure 6:**
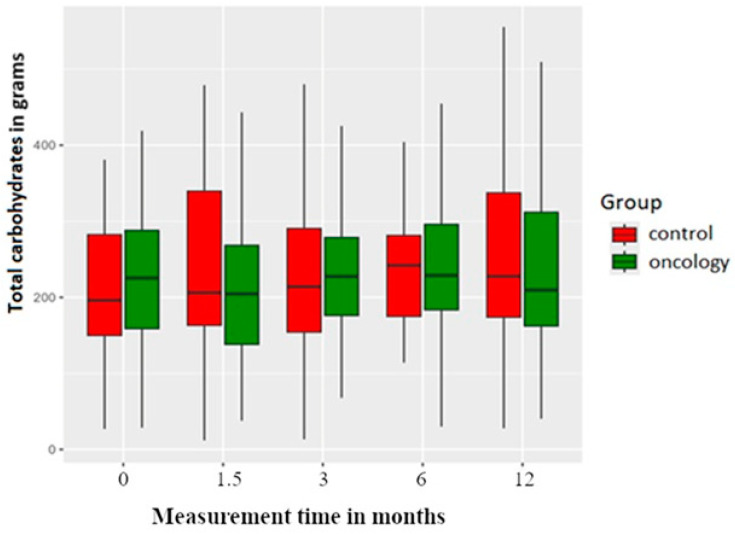
Total carbohydrates consumption in the study and control groups across all measurement periods. Time 0—before prosthetic treatment; Time 1.5—6 weeks (1.5 months) post prosthetic treatment; Time 3—3 months post prosthetic treatment; Time 6—6 months post prosthetic treatment; Time 12—12 months post prosthetic treatment.

Carbohydrate intake was higher in the study group before the treatment. Following prosthesis delivery, carbohydrate consumption was reduced in the study group at the 6-week mark but stabilised in later measurements. It was observed that carbohydrate intake increased in the control group at all measurement points after prosthesis delivery. These differences between and within both groups were not statistically significant (Figure 6).

### 3.3. Micronutrients

**Figure 7:**
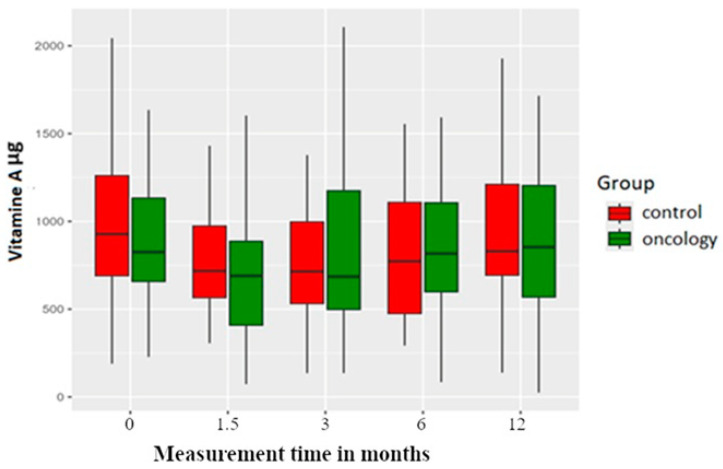
Vitamin A level in the study and control groups across all measurement periods. Time 0—before prosthetic treatment; Time 1.5—6 weeks (1.5 months) post prosthetic treatment; Time 3—3 months post prosthetic treatment; Time 6—6 months post prosthetic treatment; Time 12—12 months post prosthetic treatment.

Pretreatment Vitamin A intake was significantly lower in the study group than in the control group. Vitamin A is usually found in food items such as eggs, fish, meat, olive oil and certain vegetables. Vitamin A intake dropped for both groups in the 6 weeks post prosthesis delivery measurement but then improved in subsequent measurements. However, these differences were not statistically significant within or between the two groups (Figure 7).

**Figure 8:**
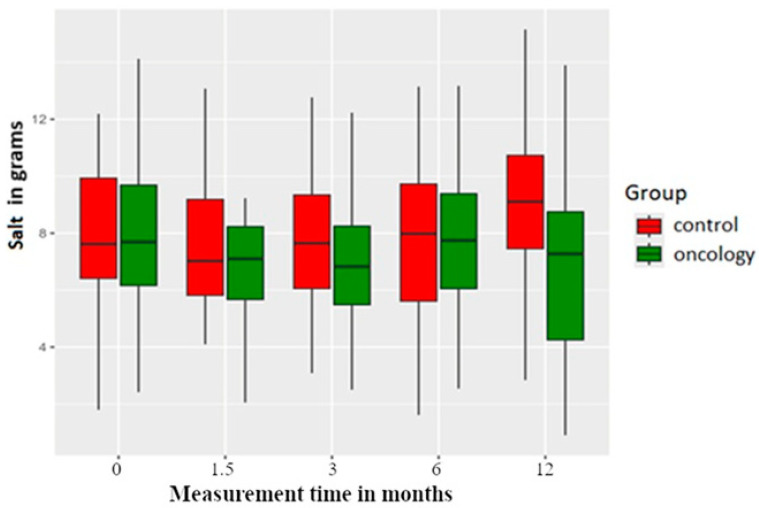
Salt intake in the study and control groups across all measurement periods. Time 0—before prosthetic treatment; Time 1.5—6 weeks (1.5 months) post prosthetic treatment; Time 3—3 months post prosthetic treatment; Time 6—6 months post prosthetic treatment; Time 12—12 months post prosthetic treatment.

Pretreatment salt consumption was similar in both groups. Post prosthesis delivery, it was found that there was a consistent reduction in salt intake in the study group compared to the control group across all the measurement points. However, these differences were not statistically significant within or between the two groups (Figure 8).

**Figure 9:**
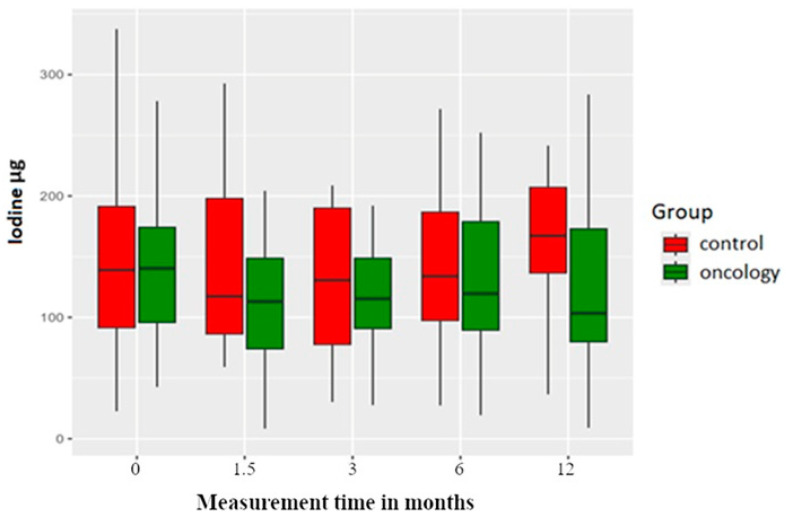
Iodine levels in the study and control groups across all measurement periods.Time 0—before prosthetic treatment; Time 1.5—6 weeks (1.5 months) post prosthetic treatment; Time 3—3 months post prosthetic treatment; Time 6—6 months post prosthetic treatment; Time 12—12 months post prosthetic treatment.

Pretreatment iodine intake was comparable between the two groups. Iodine is usually found in seafood, iodinated salt and certain vegetables. Although there was an initial drop in iodine intake at the 6-week measurement post prosthesis delivery for both groups, the control group subsequently exhibited a steady rise with a significant increase in iodine consumption at the 12-month measurement. Conversely, the study group experienced a decline in iodine intake after six weeks, with this downward trend persisting across subsequent measurements (Figure 9).

**Figure 10:**
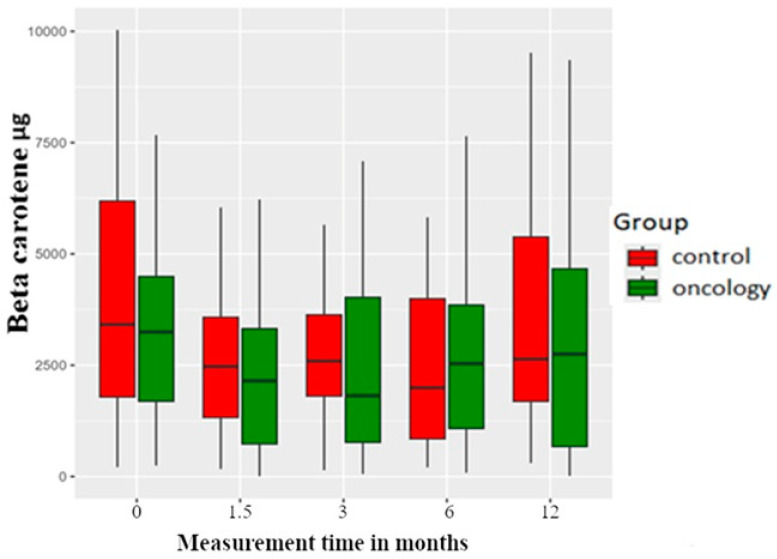
Beta carotene levels in the study and control groups across all measurement periods. Time 0—before prosthetic treatment; Time 1.5—6 weeks (1.5 months) post prosthetic treatment; Time 3—3 months post prosthetic treatment; Time 6—6 months post prosthetic treatment; Time 12—12 months post prosthetic treatment.

β-carotene pretreatment measurements revealed no difference amongst the groups. Vegetables like carrots, green pepper and spinach and fruits such as peaches and apricot are high in β -carotene. Interestingly, at post-delivery measurements, it was observed that consumption decreased initially but reached the pretreatment levels at the 12-month post-treatment measurements. However, there were no statistical differences observed within or between the groups (Figure 10).

Other elements such as sodium, potassium, calcium, phosphorus, iron and zinc were measured, and it was found that the study group’s meals contained smaller amounts compared to the control group, although the differences were not statistically significant.

### 3.4. Changes in the Study Group

In the study group, statistically significant changes were observed in the amount of total protein and animal protein consumed between 6 weeks and 3 months of wearing dentures. Statistically changes were also observed for sodium, Vitamin A, β-caroten, Vitamin B12 and Vitamin D. Statistically lower glucose and salt consumption was recorded after 3 months of wearing dentures (Table 3).

Observations regarding the frequency of consumption of certain food products that revealed statistical significance are also tabled here. The consumption of wheat and wholemeal diary products including sweet pastries were reviewed. Patients in the control group showed a higher preference for wheat ad rye bakery products and sweet pastries post prosthesis delivery as compared to before the treatment, and this was statistically significant.

### 3.5. Food Frequency

For milk and dairy product intake, it was significantly lower in the study group across all measurements. The study group and the control group tended to consume more eggs post treatment; however, this change was not statistically significant both within and between the groups. The study group was less likely to choose lean poultry such as chicken and turkey. Instead, their meat consumption included a higher proportion of organ meats (such as offal, liver, kidney) and processed meat. In comparison, the control group exhibited a higher frequency of processed meat consumption, averaging seven times a week, after 12 months of prosthesis use, although this was not statistically significant.

## 4. Discussion

With the advent of digital technologies and osseointegration in the surgical management of head and neck oncology, there is a push towards the use of fixed prostheses. Although these have been shown to improve the quality-of-life measures in most situations, this technology is not currently prevalent in most parts of the world [4,14]. Removable prostheses are still a viable option in most parts of the world due to its simplicity and affordability. A removable prosthesis does come with its advantages and disadvantages as with any other treatment modality. Literature studies have shown that removable prosthesis use can improve or make it challenging for maintaining adequate nutritional status [10,15].

This study was carried out to assess if the provision of removable prosthetic restorations to edentulous head and neck cancer patients who underwent surgical management affected their eating habits and their nutritional intake. The use of removable prosthetic restorations could cause limitations regarding chewing efficiency, progressively worsening their nutritional habits [16,17].

Generally, the results indicated that there were no significant differences within and between the study and control groups. The lack of statistically significant differences between the groups suggests that the consumption of certain nutrients between the groups may result from habits related to age, physical activity, education, social status, etc.

The consumption of main nutrients in the study group decreased in the first measurement. The energy value of meals, fat, protein and carbohydrates decreased. After a year of using dentures, we can observe an increase which means slow adaptation to the dentures. Similar observations have been reported by Moynihan P et al. for patients wearing complete dentures [10].

Of the significant differences reported were pretreatment Vitamin A intake between the study and the control group. This was challenging to explain as both groups would have had access to the food items and the same difficulty in consuming them. Vitamin A is considered to be an antioxidant. Free radical formation has been shown as a cancer causative factor [18]. Antioxidants such as β-carotene, omega-3 acids and Vitamin A provide a protective effect against cancers. It could be argued that perhaps the significant low pretreatment levels amongst the study group points to a reason for the origin of cancer in this group, although it must be stressed that this cannot be substantiated with the available information.

Vitamin A preclinical studies predominantly showed therapeutic properties through its inhibitory effects on tumour cell growth and differentiation [19,20]. However, one in vitro study demonstrated no effect on preventing the progression of oral lichen planus into oral cancer [21]. In clinical studies investigating oral potentially malignant disorders, reduction in lesion size and number as well as improvement in patient symptoms in Vitamin A adjunct or monotherapies were observed [22,23,24].

Similarly, for iodine intake, there was a significant difference between the groups at the 12-month post-delivery period. It has been shown that the control group was consuming more processed meat, and as processed meat will have more salt, it is likely that this salt would be iodinated and hence the result. Figure 8 also reveals that although there was no statistical difference between the two groups at the 12-month post-delivery period, visually, there appears to be a difference in the salt intake.

The control group did show a preference for wheat and rye bakery products and sweet pastries post prosthesis delivery. This positively showcases the improved chewing efficiency of the prosthesis for harder food products, which would have been difficult to comminute for edentulous patients.

A finding which was challenging to explain was the significant reduction in milk and dairy product consumption for the study group across all measurements. A possible explanation could be dysgeusia caused by the surgical treatment; however, this should affect all other food items as well.

Adequate nutrition, including a diet rich in fruits and vegetables, relies heavily on efficient chewing function. It has been noted that patients with compromised chewing often avoid fibrous and crunchy food, reflecting a relationship between diet quality and oral function [25]. This aligns with findings that limited food diversity and reduced intake of key nutrients such as β-carotene and omega-3 acids, along with reduced prosthesis adaptation, may hinder nutrition. The importance of adequate protein intake has been emphasised, particularly in elderly patients, to prevent undernutrition and sarcopenia [26]. Protein requirements often increase in illness and recovery. Other affects have also been linked to compromised chewing function such as gastric mucosal lesions and finding chronic gastritis in edentulous patients with impaired chewing [27].

The management of head and neck cancer patients is in a multidisciplinary setting. Several clinical guidelines advise the importance of including clinical dietitians in the multidisciplinary team to improve nutritional outcomes through dedicated support and education [28,29]. A dietitian is not part of the team managing oncology patients in Poland. Dietary preferences in the oncology group suggest the need for changes in the selection of products. This could be achieved by helping a dietitian select the right diet. The diet should complement all the ingredients and also be easy to consume.

At times, there is an implicit assumption that by having teeth, even though they are artificial in nature, oral function returns to pre-extraction levels. Patients may want to go back to eating like they used to when they had their natural dentition; however, artificial dentition does not provide one with the same occlusal comminution advantage as natural dentition does nor can it be expected to function the same way as natural dentition. Patients using removable prosthetic restorations often experience significant oral disability, which can initially worsen their nutritional habits [10,15]. Perhaps this realisation comes with time and continued use of the removable prosthesis and improves their nutritional status over time, as we see amongst the results.

This study included all patients who met the inclusion criteria and were treated in the Prosthetic Department at MUB. Due to the limited number of patients who met the criteria during this period, a total population sampling strategy was used within a single clinical centre. This means that the study included the entire available population of patients eligible for the study at a given time, rather than a random sample. It should be noted that the statistical power analysis indicates the need for a larger sample size to detect small effects. This study is observational in nature and reflects the real clinical conditions of the treatment of patients after oncological procedures at a given centre. According to the statistical power analysis, to detect the observed difference (d = 0.17) with 80% power, 274 subjects would have to be recruited to each group (study group and control group). Due to the limited availability of patients meeting the inclusion criteria, the group sizes were smaller, which may limit the power of statistical inference.

## 5. Conclusions

Although a protracted adaptation period to removable prosthetic restorations was elicited in the results, as evidenced by the initial decline in the diet after the delivery of the prostheses and improvement over time, it can be suggested that removable prostheses are a viable restoration option for surgically managed edentulous head and neck cancer patients. No statistical differences were found apart from the intake of Vitamin A and milk and dairy products amongst the groups. This study also underscores the importance of the inclusion of clinical dietitians in the multidisciplinary team to enhance nutritional education and facilitate improved dietary choices.

## Figures and Tables

**Table 1:** Inclusion criteria.

Study Arm	Control Arm
History of surgical resection due to head and neck cancer	No history of head and neck cancer
Never used a removable prosthesis	Never used a removable prosthesis
Complete edentulism	Complete edentulism
Needs and consents for prosthetic restoration	Needs and consents for prosthetic restoration

**Table 2:** Exclusion criteria.

Study Arm	Control Arm
Metabolic disease (obesity, type 1 or 2 diabetes), cardiac disease, autoimmune disease, intestinal tract disease, infectious disease (infection with HIV or HCV), immunity disorder, another neoplasm or a chronic infectious disease.	Metabolic disease (obesity, type 1 or 2 diabetes), cardiac disease, autoimmune disease, intestinal tract disease, infectious disease (infection with HIV or HCV), immunity disorder, another neoplasm or a chronic infectious disease.
Radiotherapy, chemotherapy	

**Table 3:** Comparison of macro- and micronutrients in the study group in relation to point 0.

Component	Measurement	Study Group (n = 44)							*p* *
		Average	Standard Deviation	Minimum	Q1	Median	Q3	Maximum	
Energy	0	1642.06	638.29	438.34	1196.14	1522.25	1945.12	3582.07	
	A	1502.56	534.54	286.97	1204.63	1406.18	1873.40	2848.89	0.549
	B	1605.53	668.77	418.50	1245.48	1480.61	1798.14	4189.05	0.946
	C	1709.80	616.54	274.87	1298.44	1533.71	2030.15	3278.26	0.603
	D	1758.89	705.79	236.96	1318.34	1503.89	2330.26	3335.00	0.518
Water	0	1342.01	606.36	397.17	941.67	1135.35	1794.24	2968.84	
	A	1219.84	568.15	342.25	809.57	1088.81	1596.88	2727.19	0.187
	B	1256.34	529.74	269.30	968.86	1224.63	1475.01	2801.34	0.187
	C	1345.65	662.95	426.99	792.71	1300.50	1671.97	3373.44	0.831
	D	1323.14	664.47	419.83	847.57	1164.17	1760.92	3087.89	0.831
Protein	0	74.56	25.17	23.52	59.02	73.08	84.87	156.67	
	A	68.16	22.36	14.11	53.76	61.15	87.84	116.43	0.045
	B	67.59	26.20	32.15	52.94	59.07	71.80	155.21	0.045
	C	75.20	25.69	29.74	54.67	70.20	96.53	146.53	0.764
	D	78.22	31.98	13.98	55.36	73.76	100.78	147.36	0.764
Animal protein	0	49.15	17.79	8.31	36.76	48.00	60.11	118.01	
	A	44.08	18.90	9.76	31.33	39.57	51.55	105.49	0.007
	B	42.14	20.37	12.66	30.94	37.90	46.72	123.37	0.003
	C	47.37	19.74	10.55	31.50	43.34	64.41	95.93	0.072
	D	50.98	26.12	5.40	32.36	46.97	68.96	127.10	0.590
Vegetable proteins	0	24.03	11.57	3.39	16.34	21.67	28.51	59.59	
	A	22.59	9.76	3.50	15.60	21.71	29.00	46.15	0.639
	B	23.30	13.97	0.15	17.49	22.56	26.63	94.84	0.893
	C	25.80	12.72	4.46	18.59	24.78	29.77	81.84	0.162
	D	25.09	14.42	5.28	15.83	20.39	30.77	81.90	0.639
Fat	0	47.12	19.91	19.27	30.76	41.92	65.23	104.29	
	A	44.29	23.94	9.54	26.84	39.16	54.41	114.76	0.380
	B	47.82	27.18	13.59	28.81	40.16	53.28	126.40	0.472
	C	50.16	28.60	5.43	33.32	43.50	65.81	169.24	0.590
	D	54.91	27.80	4.03	37.44	50.47	72.08	132.11	0.201
Carbohydrates	0	238.71	112.58	28.61	155.35	225.38	302.49	601.20	
	A	216.04	105.82	37.80	130.65	204.50	273.43	509.52	0.471
	B	235.18	113.82	13.35	174.76	227.49	284.10	649.35	0.840
	C	248.83	108.99	29.98	182.01	228.82	297.88	499.08	0.471
	D	247.43	129.13	40.36	156.55	209.63	322.14	509.52	0.741
Sodium	0	3229.46	1279.82	966.55	2453.14	3075.65	3962.52	6979.30	
	A	2876.49	1447.72	438.02	2116.94	2837.22	3299.93	9218.69	0.056
	B	2898.94	1408.63	248.50	2200.90	2728.50	3372.56	7934.37	0.015
	C	3179.75	1198.37	1016.36	2412.80	3114.67	3798.16	7109.75	0.893
	D	3023.89	1703.07	362.15	1728.29	2981.47	3657.61	9218.69	0.191
Potassium	0	3173.72	1874.41	689.06	1888.20	2368.16	3909.33	8344.55	
	A	2718.59	1342.45	518.65	1745.58	2554.15	3518.04	7325.31	0.615
	B	3352.59	2449.83	778.00	2061.45	2627.27	3750.18	14,696.06	0.893
	C	3299.51	1758.04	1024.41	2172.54	3025.85	3903.88	9242.59	0.615
	D	3289.35	1859.39	1363.78	1923.69	2961.74	3887.09	9213.08	0.615
Calcium	0	660.38	326.28	163.70	419.07	596.31	857.82	1631.63	
	A	548.13	294.43	82.86	345.08	530.24	677.57	1313.32	0.128
	B	598.11	319.99	110.24	325.53	503.03	805.48	1292.78	0.360
	C	626.48	310.33	147.69	393.99	540.28	861.74	1354.74	0.360
	D	671.39	384.06	84.05	401.88	609.60	886.21	2139.97	0.567
Phosphorus	0	1247.59	480.62	444.77	968.00	1142.34	1457.96	2348.17	
	A	1096.57	459.56	228.95	803.77	1032.95	1368.22	2567.58	0.109
	B	1273.12	773.79	582.00	795.38	1016.04	1515.80	5018.15	0.662
	C	1290.08	605.63	322.91	942.26	1102.79	1500.30	4076.43	0.809
	D	1346.50	693.98	360.04	952.72	1180.03	1521.44	4007.28	0.809
Magnesium	0	262.08	118.57	55.65	171.66	241.24	312.39	586.17	
	A	247.49	111.34	43.91	175.30	222.13	306.39	532.11	0.786
	B	263.36	157.17	69.00	184.10	240.44	285.90	1110.71	0.786
	C	284.00	139.97	54.92	205.32	263.21	330.53	973.21	0.591
	D	283.00	157.32	99.60	169.91	249.55	370.31	973.11	0.591
Iron	0	10.58	5.20	2.87	6.99	8.67	13.60	23.77	
	A	9.56	5.23	1.39	5.89	8.66	11.04	27.43	0.174
	B	11.51	9.40	2.10	7.35	9.66	12.07	63.49	0.736
	C	11.72	7.91	2.45	7.64	9.53	13.41	52.91	0.707
	D	727.38	4748.16	2.48	7.59	8.72	15.11	31,507.25	0.707
Zinc	0	8.88	3.56	3.05	6.71	8.01	10.60	21.19	
	A	8.38	3.56	1.71	6.05	7.73	9.89	17.84	0.425
	B	8.49	4.18	2.81	6.33	7.60	8.70	27.46	0.425
	C	9.41	4.33	2.35	6.82	8.37	10.98	27.49	0.425
	D	9.24	4.54	2.02	6.41	8.10	11.17	26.46	0.973
Copper	0	1.07	0.55	0.21	0.71	0.90	1.36	2.48	
	A	1.06	0.62	0.20	0.65	0.93	1.32	3.40	0.672
	B	1.15	0.60	0.46	0.84	1.00	1.24	3.32	0.672
	C	1.17	0.54	0.13	0.86	1.08	1.33	2.53	0.495
	D	1.18	0.65	0.27	0.73	1.01	1.41	3.57	0.495
Manganese	0	3.40	1.87	0.36	2.11	3.12	4.55	9.04	
	A	3.57	2.50	0.30	1.98	3.05	4.27	13.12	0.461
	B	3.57	1.90	0.12	1.91	3.54	4.86	9.89	0.515
	C	3.86	1.51	1.43	3.02	3.84	4.54	7.81	0.403
	D	3.73	2.31	0.67	1.96	3.39	4.21	13.12	0.515
Vitamin A	0	958.88	535.42	227.86	649.13	824.99	1145.42	3250.81	
	A	757.12	507.28	72.91	406.02	689.61	904.24	3185.59	0.007
	B	873.02	566.58	135.50	478.18	685.39	1190.31	2795.60	0.007
	C	888.08	461.74	84.78	593.73	817.00	1109.12	2293.61	0.112
	D	973.52	616.56	24.93	559.52	853.60	1252.14	2672.49	0.544
Retinol	0	334.25	131.49	107.19	231.83	337.04	426.07	595.31	
	A	349.90	477.98	38.42	165.90	233.83	339.87	3092.28	0.216
	B	451.47	513.47	68.62	183.86	312.81	439.35	2593.43	0.412
	C	337.03	201.14	28.37	183.33	341.58	453.03	897.48	0.946
	D	415.32	327.82	21.00	208.15	369.06	528.80	1732.54	0.216
Beta-carotene	0	3607.64	3030.18	245.52	1537.17	3247.70	4588.69	16,875.82	
	A	2290.56	1786.62	7.67	717.46	2149.59	3402.52	7259.28	0.004
	B	2393.56	1941.22	56.56	746.64	1815.53	4046.03	7082.93	0.004
	C	3171.01	2723.27	84.40	1062.66	2536.22	3906.33	13,279.39	0.243
	D	3222.94	3078.22	14.02	635.04	2749.50	4763.68	13,366.49	0.243
Vitamin E	0	4.98	2.56	2.03	3.30	4.59	5.88	16.72	
	A	4.68	2.75	1.00	2.99	3.93	5.92	15.59	0.710
	B	4.95	3.41	1.15	3.64	4.49	5.38	23.82	0.913
	C	4.97	3.10	0.62	3.43	4.23	5.36	20.94	0.913
	D	5.64	4.10	1.72	3.34	4.27	6.70	21.78	0.868
Thiamine	0	1.05	0.54	0.20	0.70	0.89	1.40	2.59	
	A	0.97	0.49	0.19	0.72	0.90	1.10	2.31	0.711
	B	1.02	0.61	0.10	0.63	0.89	1.27	3.04	0.695
	C	1.10	0.52	0.16	0.79	0.96	1.40	2.56	0.248
	D	1.12	0.60	0.25	0.64	0.98	1.51	2.31	0.248
Riboflavin (B2)	0	1.58	0.58	0.56	1.13	1.53	1.84	2.99	
	A	1.40	0.55	0.40	1.00	1.36	1.80	2.77	0.092
	B	1.55	0.61	0.54	1.06	1.39	1.95	3.29	0.893
	C	1.58	0.58	0.53	1.12	1.54	1.88	3.12	0.868
	D	1.59	0.63	0.55	1.01	1.58	1.91	3.06	0.893
Niacin (B3)	0	18.05	11.67	3.27	9.88	14.74	22.19	51.91	
	A	14.83	7.51	1.94	10.44	13.73	17.28	43.42	0.340
	B	19.13	16.77	3.50	9.09	14.53	22.32	91.37	0.707
	C	18.59	11.75	6.45	10.13	14.78	22.00	50.82	0.893
	D	19.24	11.93	2.35	9.85	14.71	26.10	51.54	0.707
Vitamin B6	0	1.70	0.79	0.66	1.05	1.54	2.13	3.84	
	A	1.54	0.71	0.22	1.05	1.49	1.88	3.45	0.381
	B	1.71	0.88	0.54	1.05	1.54	2.35	5.01	0.893
	C	1.77	0.76	0.34	1.18	1.74	2.39	3.23	0.850
	D	1.76	0.81	0.57	1.01	1.73	2.40	3.68	0.873
Vitamin C	0	74.20	79.43	5.44	33.72	47.52	91.51	370.20	
	A	72.91	73.11	10.13	22.17	47.05	97.19	352.35	0.591
	B	65.75	70.70	2.85	22.03	49.75	78.49	372.06	0.591
	C	73.52	78.22	8.72	31.34	45.33	94.22	371.04	0.711
	D	74.17	68.84	6.22	32.01	53.61	93.93	358.79	0.591
Total saturated fatty acids	0	20.58	10.17	5.48	12.73	17.84	28.39	51.08	
	A	20.39	12.90	4.41	11.86	17.08	27.12	74.11	0.380
	B	21.42	14.23	4.41	11.92	17.13	27.14	68.56	0.380
	C	21.42	14.45	2.06	11.97	19.18	25.90	81.57	0.813
	D	25.11	15.32	0.77	14.90	24.04	31.21	82.43	0.187
Monosaturated fatty acids	0	15.59	6.70	4.21	10.73	13.57	19.40	31.47	
	A	13.81	9.49	3.65	8.54	10.38	15.79	48.49	0.118
	B	14.83	7.96	3.78	9.90	12.39	18.19	32.00	0.472
	C	16.56	9.97	1.55	10.68	14.06	20.28	62.83	0.472
	D	17.48	9.18	0.84	11.24	16.15	24.36	34.93	0.306
Polyunsaturated fatty acids	0	6.20	4.50	0.90	3.56	5.01	6.92	24.73	
	A	5.76	4.55	0.80	3.38	4.29	7.01	24.93	0.866
	B	6.39	7.54	1.02	3.67	4.39	6.40	49.97	0.786
	C	7.02	7.64	1.02	3.65	5.31	7.16	49.74	0.301
	D	6.79	7.59	0.47	3.37	5.42	6.85	49.88	0.483
Cholesterol	0	297.72	144.92	69.04	176.23	288.39	377.67	696.20	
	A	231.13	118.61	68.71	144.29	189.13	325.97	637.21	0.248
	B	264.56	182.62	28.80	147.50	217.77	320.84	1053.77	0.273
	C	292.32	172.75	72.83	159.55	225.05	419.99	717.54	0.973
	D	291.12	159.07	12.00	160.35	228.15	414.51	660.05	0.431
Saccharose	0	31.45	20.30	1.26	16.08	29.72	46.32	78.19	
	A	30.73	25.12	1.43	10.20	25.70	36.76	99.15	0.853
	B	31.39	28.44	1.88	11.33	24.91	41.35	140.05	0.853
	C	34.59	32.79	2.53	15.00	24.79	42.93	149.64	0.853
	D	35.25	25.48	2.52	14.52	30.85	53.34	100.05	0.853
Lactose	0	9.17	7.12	0.06	3.24	8.02	13.36	32.94	
	A	8.65	7.31	0.05	2.10	7.25	13.54	26.05	0.919
	B	9.24	7.15	0.00	2.96	6.73	15.24	30.34	0.495
	C	8.02	6.41	0.00	1.92	7.90	12.34	25.04	0.831
	D	9.28	7.44	0.00	2.46	8.81	14.87	27.83	0.495
Starch	0	134.58	63.91	18.04	94.73	123.69	152.43	340.94	
	A	125.70	60.27	15.76	84.15	114.68	161.33	273.35	0.425
	B	119.65	49.99	0.00	100.82	128.62	150.01	214.06	0.571
	C	140.07	72.57	6.58	97.04	134.58	166.44	426.73	0.571
	D	129.84	70.83	6.79	81.25	115.77	179.78	308.19	0.571
Fibre	0	17.73	9.83	2.09	11.11	15.03	24.36	41.19	
	A	16.57	8.95	2.74	9.74	15.56	21.97	45.00	0.764
	B	18.27	13.28	0.45	10.25	15.96	24.22	83.84	0.764
	C	18.98	10.28	4.66	13.24	16.80	23.54	64.88	0.265
	D	19.13	11.53	5.81	12.06	15.53	22.21	64.88	0.483
Folates	0	233.26	113.67	50.47	166.23	211.59	267.46	605.01	
	A	209.96	107.72	61.35	127.16	193.07	255.49	641.71	0.265
	B	226.35	103.69	17.95	178.15	206.11	260.38	648.70	0.840
	C	239.15	96.38	72.03	179.22	227.16	273.99	656.63	0.380
	D	4294.88	26,887.92	102.87	164.28	212.97	310.52	178,594.35	0.558
Vitamin B12	0	3.76	2.02	0.52	2.29	3.57	4.36	10.57	
	A	2.92	1.68	0.51	1.73	2.59	3.65	8.31	0.018
	B	4.06	4.86	0.87	1.69	2.84	4.17	26.53	0.227
	C	3.17	1.33	1.14	1.99	2.97	4.32	6.32	0.786
	D	3.36	1.60	0.75	2.15	3.31	4.22	8.31	0.786
Vitamin D	0	3.17	2.95	0.17	1.34	2.05	3.34	13.50	
	A	1.95	2.00	0.21	0.80	1.35	2.24	10.72	0.018
	B	3.36	6.17	0.08	0.86	1.69	2.88	38.02	0.066
	C	2.53	1.95	0.19	1.20	2.26	3.07	10.73	0.741
	D	3.24	3.38	0.05	1.22	2.19	3.60	12.37	0.741
Iodine	0	147.18	65.55	42.64	94.18	140.39	176.91	360.65	
	A	117.06	64.06	8.38	73.21	113.02	154.63	344.39	0.004
	B	124.82	63.41	2.76	87.63	115.33	155.00	329.44	0.004
	C	135.89	70.38	19.49	87.01	119.59	181.67	350.21	0.106
	D	127.59	77.66	9.08	79.04	103.40	173.92	384.95	0.091
LCPUFAs	0	0.28	0.54	0.00	0.03	0.08	0.15	2.73	
	A	0.15	0.34	0.00	0.02	0.04	0.11	2.03	0.741
	B	0.22	0.56	0.00	0.02	0.05	0.10	2.78	0.440
	C	0.15	0.29	0.00	0.02	0.07	0.14	1.50	0.741
	D	0.23	0.43	0.00	0.04	0.08	0.13	2.03	0.128
Digestible carbohydrates	0	220.98	105.16	26.53	143.09	210.06	289.39	561.93	
	A	199.47	99.31	34.42	120.91	192.83	248.42	464.52	0.425
	B	216.91	103.27	12.90	162.59	210.55	262.44	565.50	1.000
	C	229.85	103.12	25.32	167.36	211.23	272.83	489.84	0.425
	D	228.30	121.02	31.68	148.56	197.12	301.35	468.66	0.873
Folic acids	0	7.55	45.38	0.00	0.00	0.00	0.00	300.00	
	A	8.64	46.54	0.00	0.00	0.00	0.00	300.00	0.987
	B	9.99	46.71	0.00	0.00	0.00	0.00	300.00	0.987
	C	8.89	46.52	0.00	0.00	0.00	0.00	300.00	0.987
	D	2395.77	15,827.96	0.00	0.00	0.00	0.00	105,000.00	0.987
Omega 3 fatty acids	0	1.52	2.70	0.13	0.53	0.66	1.42	15.62	
	A	1.28	2.62	0.13	0.42	0.60	1.01	16.53	0.448
	B	1.78	5.13	0.16	0.42	0.72	1.10	34.05	0.637
	C	1.76	5.10	0.07	0.54	0.75	1.04	33.94	0.448
	D	1.85	5.13	0.21	0.54	0.73	1.07	33.99	0.448
Omega 6 fatty acids	0	4.39	2.14	1.40	2.95	3.60	5.46	10.01	
	A	4.10	2.50	0.62	2.74	3.56	4.95	15.14	0.893
	B	4.22	2.47	0.43	2.83	3.71	5.36	15.91	0.893
	C	4.85	2.89	0.53	3.12	4.23	5.68	15.83	0.381
	D	4.62	2.70	0.24	2.85	4.42	5.68	15.89	0.617
Glucose	0	7.36	6.52	0.42	2.21	6.24	9.45	30.09	
	A	6.49	6.69	0.33	1.76	3.99	10.02	26.46	0.045
	B	6.59	6.91	0.00	1.47	4.89	9.48	35.87	0.045
	C	7.01	7.64	0.08	2.10	5.19	8.93	36.81	0.189
	D	6.49	6.06	0.00	1.88	5.31	9.27	24.56	0.084
Fructose	0	9.23	7.97	0.55	2.48	7.88	12.07	32.49	
	A	8.41	8.35	0.35	2.09	4.59	13.35	27.17	0.265
	B	8.92	8.83	0.00	1.56	6.70	13.09	39.39	0.265
	C	7.94	7.64	0.08	2.30	6.43	9.65	32.41	0.265
	D	8.65	8.27	0.00	1.89	7.00	11.55	28.39	0.266
Salt	0	8.08	3.20	2.42	6.14	7.69	9.92	17.46	
	A	7.20	3.62	1.10	5.30	7.10	8.25	23.05	0.066
	B	7.22	3.54	0.62	5.47	6.83	8.44	19.84	0.012
	C	7.93	3.00	2.54	6.04	7.74	9.50	17.78	0.893
	D	7.36	4.31	0.91	4.21	7.28	8.95	23.05	0.097

0—before prosthetic treatment. A—6 weeks after treatment. B—3 months after treatment. C—6 months after treatment. D—12 months after treatment. Red colour of result—statistically significant result. * *p* value relative to measurement 0.

## Data Availability

The raw data supporting the conclusions of this article will be made available by the authors on request.
